# The US/Mexico Border: A Binational Approach to Framing Challenges and Constructing Solutions for Improving Farmworkers’ Lives

**DOI:** 10.3390/ijerph9062159

**Published:** 2012-06-07

**Authors:** Cecilia Rosales, Maria Isabel Ortega, Jill Guernsey De Zapien, Alma Delia Contreras Paniagua, Antonio Zapien, Maia Ingram, Patricia Aranda

**Affiliations:** 1 Mel & Enid Zuckerman College of Public Health, University of Arizona, 714 East Van Buren, Phoenix, AZ 85006, USA; Email: dezapien@email.arizona.edu (J.G.D.Z.); maiai@email.arizona.edu (M.I.); 2 Nutrition Division, Research Center for Food and Development, Victoria Road, Km 0.6, La Victoria Town, 83304, Hermosillo, Mexico; Email: iortega@ciad.mx (M.I.O.); acontreras@ciad.mx (A.D.C.P.); 3 Center for Studies on Health and Society, The Sonora College, 54 Obregon Ave., Downtown Hermosillo, 83000, Hermosillo, Mexico; Email: antoniozs@msn.com (A.Z.); pag@colson.edu.mx (P.A.)

**Keywords:** jornaleros, farmworkers, migration, binational collaboration, US Mexico, border region

## Abstract

Mexican migrant and seasonal farmworkers in the US-Mexico border region face health hazards and occupational risks and are becoming commonly known in the public health literature. According to several studies, farmworkers have high levels of chronic diseases such as diabetes and respiratory problems, are at risk for infectious diseases, and experience among the highest incidences of work-related injuries of any profession. The findings from two studies are considered and presented with the objective of contributing to an overall understanding of migrant farmworkers as a workforce moving across national boundaries and affected by the work environments and health stressors both shared and unique to each context. We propose a binational approach to comprehensively address the health problems and socioeconomic challenges faced by migrant and seasonal farmworkers. In this paper we present the results of two distinct but complementary studies of farmworker health on the Arizona-Sonora border.

## 1. Introduction

The health hazards and occupational risks faced by Mexican migrant and seasonal farmworkers in the US-Mexico border region are becoming commonly known in the public health literature. Studies have found that farmworkers have high levels of chronic diseases such as diabetes and respiratory problems, are at risk for infectious diseases, and experience among the highest incidences of work-related injuries of any profession [1]. On the US side of the border, factors documented as contributing to these health problems include labor-intensive nature of the work, poor diet, pesticide exposure, lack of access to preventive health care and treatment, and poor living conditions [2,3]. Environmental stressors such as long work days, lack of job security and family separation also have a negative effect on farmworkers’ physical and emotional wellbeing [4,5,6]. Dangerous and unjust working conditions of migrant farmworkers (*jornaleros agrícolas)* in northern Mexico who earn salaries seven to nine times less than those in the United States cause even greater health risks for this population [7]. In both countries farmworkers are marginalized from basic services such as health care. In the US the National Agricultural Workers Survey found that less than a fourth of farmworkers have health insurance (23%) and only 12% have insurance through an employer [8]. In Mexico, studies have shown that less than 50% of farmworkers are covered under the Seguro Social [9,10].

A 2003 survey in the state of Sonora found that the demographic characteristics and transitory patterns of *jornaleros agrícolas*, most of whom live elsewhere in Mexico and migrate to the country’s northern states for the agricultural season, had shifted considerably from the time of a previous survey in 1998 [7]. Along the US Mexico border region, Mexican migrant and seasonal farmworkers are a binational population affected by social and political conditions in Mexico and the US International economic factors clearly affect farmworkers on both sides of the border. Data from Mexico illustrates that realities of farmworker life within the country mirror those of migrant farmworkers in the U. S. Health risks, however, could differ due to the health agendas of each country and the socioeconomic challenges of working in Mexico *versus* the US.

On the other hand, temporary migrant workers in Mexico are frequently the same population that migrates to the US. Consequently, a binational approach is needed in order to comprehensively address the health problems and socioeconomic challenges faced by migrant and seasonal farmworkers. Improved policies can establish farmworker health and safety protections as well as address the social and political factors affecting their lives.

In this paper we present the results of two distinct but complementary studies of farmworker health on the Arizona-Sonora border. The Arizona-Sonora border is an important region for the agricultural industry due to the climatic conditions that allow for a vital winter growing season. Arizona’s four border counties, Yuma, Santa Cruz, Pima and Cochise, share a southern border some 350 miles long with the Mexican state of Sonora. Yuma County is known as the lettuce capital of the nation, and lettuce is Arizona’s leading cash crop. Other major crops include broccoli, cauliflower, citrus and melons. Yuma County has the largest number of farmworkers in the state, and it is estimated that Yuma employs about half of the farmworker population. Sonora is also the home to large scale production of fruits and vegetables that are produced for export to the United States and Europe. 

Findings from these two studies are considered with the objective of contributing to overall understanding of migrant farmworkers as a workforce moving across national boundaries and affected by the work environments and health stressors both shared and unique to each context. This comprehensive picture will allow us to create a profile of migrant farmworkers in the US-Mexico border region and provide recommendations for binational programs, practices, and policies that can greatly improve the health and wellbeing of this marginalized and underserved population.

## 2. Study 1: Challenges to Farmworker Health at the US-Mexico Border

### 2.1. Methods

This US based study was a cross-sectional, population based survey conducted from August 2006 to February 2007 using a randomized proportionately representative household sample of farmworkers in the three communities of Somerton, Gadsden, and San Luis located along the US Mexico border in southern Yuma County, Arizona. The study was implemented by a multidisciplinary research team from the University of Arizona in partnership with a local farmworker agency, Campesinos Sin Fronteras. Funding was provided by the National Institute of Occupational Health and Safety. 

Households were identified using community maps and randomly selected census blocks. Standardized Spanish instruments were developed collaboratively based on reliable and validated surveys used by the California Hired Farmworker Health Survey in the California Agricultural Workers Health Survey (CAWHS) [1] that address aspects of agricultural labor and work related injury and illness. Health behavior questions were drawn from the Centers for Disease Control and Prevention’s Behavioral Risk Factor Surveillance System Survey (BRFSS) [11]. Additional questions were added to capture culturally-specific stressors, acculturation and ethnic identity. Survey areas of particular interest were stress and depression, chronic illness, musculoskeletal ailments, work-related injuries, and access to health and social services. Depression questions included the standard BFRSS questions, as well as the RAND depression questions [12]. Stress questions were devised to measure stress related to border and migration, acculturation, access to health care, economic strains and concerns for family. 

#### 2.1.1. Participant Eligibility

The survey was conducted door-to-door. Individuals were identified as farmworkers prior to being asked to participate in the study. Interviewers articulated the purpose of their visit, stated they were conducting an anonymous survey of farmworkers in the Yuma area and asked if someone in the household was over 21 years of age and working or had worked in the fields during the past year. If someone in the household met the criteria, they were invited to participate in the interview. If more than one adult in the household met the eligibility criteria, the interviewer would list in descending order of age, each man on one list, and each woman on another. Beginning with males, the interviewer would choose the one with the birth date closest to the interview date. If there were no males present in the household, then the woman with the birth date closest to the interview date would be invited to participate. 

#### 2.1.2. Interviewer Training

*Campesinos Sin Fronteras* (CSF), a key collaborator and community-based organization focused on farmworker health issues in Yuma County, Arizona, was subcontracted by the University of Arizona (UA) to hire and supervise interviewers and provide oversight of the data collection process. To describe and compare farmworker populations in a border context and from a binational perspective data analysis consisted primarily of frequency distribution. Preliminary analysis of the survey (presented below) resulted in the need for more in-depth understanding of how respondents defined stress. Group interviews were subsequently conducted to further investigate significant themes, particularly regarding stress in the context of border residence as a farmworker or farmworker family member. Four group interviews were held with farmworkers recruited by *Campesinos Sin Fronteras.* The first consisted of farmworker men of over 40 years of age, the second of farmworker women over 40, the third of farmworker men and women between 18 and 30 years of age, and the final group consisted of male and female members of farmworker families who were not farmworkers themselves. The interviews were conducted in Spanish and lasted approximately 90 minutes. 

### 2.2. Results

*Demographics*: *Campesinos* collected 298 household surveys and obtained an overall response rate of 92.7%, reflecting, we believe, the level of trust that *Campesinos* staff have established in the farmworker community. Similar to farmworker demographics, more men than women were interviewed (see Table 1) and 78% reported being married. The median age of farmworkers surveyed was 34 years of age. Most participants were male, married and of low income. On average, farmworkers had a sixth grade education, which is characteristic and consistent with findings from other studies of agricultural workers [13]. Moreover, 59–81% of those interviewed are living below the 100% federal poverty level (Table 1). In the household survey, 56.7% of those employed as farmworkers in the Yuma County area reported their place of birth as the Mexican border, while 39.8 were from other states in Mexico. A small percentage (3.5%) was reportedly born in the US. Three of four farmworkers (76.7) were legal permanent residents and 10% are naturalized US citizens. When comparing male and female respondents, women were more often single (16.9% *vs.* 7.1%), slightly younger, and were slightly more educated (67.1% of men had less than 6 years of school *versus* 76.3% of women). However, male and female farmworkers were similar in terms of place of birth and immigration status. Approximately half both male (55.2%) and female (51.7%) respondents had health insurance and this was most likely to be from the US.

**Table 1:** Arizona Demographics.

Gender	Male n % 156 (52.0)	Female n % 142 (48.0)	Total N % 298 (100)
Age			
20-34 years	21 (13.5)	31 (21.3)	52 (17.2)
35-44	44 (28.2)	59 (41.8)	103 (34.7)
45-54	52 (33.3)	37 (26.2)	89 (30.0)
55-64	28 (17.9)	15 (10.6)	43 (14.5)
65 and older	11 (7.1)	0 (0)	11 (3.7)
Marital Status			
Married	138 (88.5)	100 (70.4)	238 (79.9)
Single	11 (7.1)	24 (16.9)	35 (11.6)
Other	6 (3.8)	17 (12.0)	23 (7.7)
Missing	1 (0.064)	1 (0.70)	2 (0.67)
Birthplace			
Mexico Border	88 (56.4)	70 (49.3)	158 (53.0)
Mexico Other	62 (39.8)	67 (47.2)	129 (43.2)
US	6 (3.8)	5 (3.5)	11 (3.8)
Family Income			
<$10,000	10 (6.4)	15 (10.5)	25 (8.3)
<$15,000	25 (16.1)	28 (19.6)	53 (18.0)
<$20,000	43 (27.6)	30 (21.0)	72 (24.3)
<$25,000	47 (30.1)	30 (21.0)	77 (25.7)
<$35,000	18 (11.1)	30 (21.0)	48 (16.0)
<$50,000	9 (4.1)	7 (4.9)	14 (4.7)
<$75,000	4 (2.6)	1 (0.7)	9 (0.3)
Missing		1 (0.70)	
Education			
<6th grade	119 (76.3)	96 (67.1)	215 (71.9)
7th-9th grade	25 (16.0)	36 (25.2)	61 (20.4)
10th-12th	11 (7.7)	10 (7.7)	23 (7.7)
Missing	1 (0.064)		
Immigration Status			
Naturalized US	13 (8.4)	16 (11.5)	29 (10.0)
LPR	117 (75.0)	111 (78.3)	228 (76.7)
Other	26 (16.6)	15 (10.2)	41 (13.3)
Insurance			
US	67 (42.9)	55 (38.7)	122 (40.9)
Mexico	10 (7.0)	9 (6.3)	19 (6.3)
Both	11 (7.1)	11 (7.7)	22 (7.3)

*Health*: When asked to rate their health status (see Table 2), the majority rated their health as good (45.6%) or very good (35.2%). Rates of diagnosed chronic illness however, were very high with 16.1% diabetes, 21.5% hypertension, 12.1% arthritis and 5.4% asthma. Men reported higher rates of diabetes, hypertension and arthritis. The rate of anemia among women was 14.1%. Musculoskeletal ailments were also very prevalent, not surprising considering the intensive, repetitive and physically demanding nature of agricultural labor. Back pain was most prominently noted (35.2%), as well as hands (28.5%), feet (31.2%), and knees 26.87%). Musculoskeletal pain was high among both men and women, although women reported more hand and feet pain and men more back pain. A perhaps more unexpected finding is the high prevalence of depression, especially among women. One in four women (25.7%) reported experiencing depression for more than 2 weeks in the previous 12 months, compared to 13.5% of men.

**Table 2:** Arizona Farmworker Health N = 298.

Gender	Male n % 156 (52.0)	Female n % 142 (48.0)	Total N % 298 (100)
Self rated health status			
Poor	7 (4.5)	7 (4.9)	14 (4.7)
Fair	21 (14.5)	15 (10.6)	36 (12.1)
Good	65 (41.6)	71 (50.0)	136 (45.6)
Very Good	60 (38.5)	48 (33.8)	108 (35.2)
Excellent	3 (1.9)	1 (0.7)	4 (1.3)
Diagnosed Chronic illness			
Diabetes	33 (21.2)	15 (10.6)	48 (16.1)
Hypertension	38 (24.4)	26 (18.3)	64 (21.5)
Arthritis	22 (14.1)	14 (9.9)	36 (12.1)
Asthma	8 (5.1)	8 (5.6)	16 (5.4)
Anemia	1 (0.6)	20 (14.1)	21 (7.0)
Musculoskeletal injury			
Back pain	61 (39.1)	44 (30.9)	105 (35.2)
Neck pain	43 (27.5)	24 (16.9)	67 (22.4)
Knee pain	46 (29.5)	34 (23.9)	80 (26.8)
Shoulder pain	34 (21.8)	24 (16.9)	58 (19.5)
Hand pain	38 (24.4)	47 (33.1)	85 (28.5)
Feet pain	43 (27.5)	50 (35.2)	93 (31.2)
Depression	21 (13.5)	38 (25.7)	59 (19.7)
Nutrition (Servings per day)			
Vegetables	1.8	1.7	1.8
Fruit	1.6	2.0	1.8
Flour tortillas	3.8	1.6	2.7
Corn tortillas	5.0	3.9	4.5

Additionally, farmworkers reported experiencing stress related to their migrant status, their foreign/racial minority position, and the current political environment. Seventy-three percent (73%) cited the need to speak better English as a significant stressor and 41% experienced stress related to traveling far from their families to earn a living. Over 40% experienced stress associated with obtaining health services for their families, and many worried about supporting themselves and their families in the long term. Together with economic worries, farmworkers reported stress tied to a climate of fear or oppression in their border community. Stressors also included difficulty in getting ahead in the US due to their race, encounters with police and border patrol officials, the construction of the border wall, and the unjust treatment of others of their same racial and ethnic background. 

Focus group findings expanded on survey results with respect to stress. Along with stress related to lack of English skills and family separation, many also described stress caused by abusive or discriminatory interactions with officials from Customs and Border Protection and Border Patrol. Younger focus group participants in particular reported incidents of abuse. Moreover, participants reported work-related stress, including abuse from foremen and supervisors, as well as stress associated with agricultural work itself. Farmworkers in the focus group expressed a need for better working conditions, including higher salaries, health insurance and related benefits, opportunities to develop their skills, and prospects for improving English proficiency.

### 2.3. Study Limitations

Although the two studies are mainly descriptive, the mixed methods approach strengthened our discussion. Enrollment of workers in study 2 was voluntary and in accordance with the international Review Board policies for research with human subjects, the research team observed the non-discriminatory and voluntary participation policies. Sampling these migrant populations is challenging given they are constantly migrating between regions and farms. Moreover, they live and work in private enterprises whose owners are often reluctant to participate in studies investigating the living conditions of their workers. Accounting for these sampling conditions we can assume that there must be a sampling bias that is inherent to a very mobile population.

Enrollment of workers in study one was voluntary in accordance with IRB policies for research with human subjects. Interviewers were local community health workers (*promotores*) who had been farmworkers themselves and knew the community quite well, resulting in a high response rate. However, the climate of fear due to the militarization of the border may have contributed to workers focusing more on those issues as stressors rather than some of the occupational stressors. Additionally border farmworker communities have unique issues related to being situated in a binational region and thus some of the findings may not be generalizable to farmworker communities in other areas of the country. 

## 3. Study 2: The Health of Migrant Farmworkers and a Model of Social Responsibility in Agribusiness in Sonora

The second study of migrant farmworkers in northern Mexico has provided an important opportunity for understanding some of the demographic and health characteristics of this population. A cross-border interdisciplinary research team surveyed 233 workers in agricultural labor camps located in the state of Sonora, and conducted 17 group and key informant interviews creating a demographic and health profile of the migrant population. The team included two sociologists, a physician and public health researcher, two nutritionists, an agronomist, a communication specialist and two public health professionals, from three different academic institutions in the US and Mexico (University of Arizona College of Public Health, El Colegio de Sonora [COLSON] and Centro de Investigación en Alimentación y Desarrollo [CIAD]). El Colegio de Sonora, CIAD, and UA research staff are all trained interviewers who have worked collaboratively on a number of other studies in the past. All interviewers/researchers were required to pass the Rochester Exam and provided training on data collection, confidentiality, and privacy issues. This training included an overview and point by point review of ethical interviewing practices, the anonymous nature of the survey, non-disclosure of respondents’ identities, addresses or any other identifiers under any circumstances, and the confidentiality of all interview content. Role plays were utilized in the training when appropriate. The study included a convenience sample collected on farms in Sonora between April and November of 2007. Funding was provided by the binational *Programa de Investigacion en Migracion y Salud* (PIMSA). 

Agribusiness in Sonora consists of private businesses that require the owner’s permission to speak to workers who live on the farm premises. Three farm owners provided permission to visit their installations and talk to workers. A team of nine interviewers contacted each *jornalero* or *jornalera* who was living on the farm to ask for permission to be interviewed. Interviews were conducted on a total of 17 different days between April and November of 2007, for a total of 233 interviews. The interview questions were derived from instruments developed in the United States including the National Agricultural Workers Survey, the California Agricultural Workers Health Study [8], and the Binational Farmworker Health Survey [14] and from instruments developed in Mexico by the Mexican Secretariat of Social Development (SEDESOL) and the National Agricultural Farmworker Program [7]. The questions addressed aspects such as demographics, working and living conditions, health status, health services and governmental aid program access, as well as training regarding personal hygiene, pesticide management and food safety. Workers were approached to participate in interviews during packing in the fields, rest periods and after work. The interviews which were conducted with individuals lasted between 15 to 30 minutes. 

In addition, a trained facilitator conducted nine group interviews, three per farm, including between two and five *jornaleros* per group and eight key informant interviews with farm owners and farm managers. A guide was used to conduct group discussions. The discussions lasted 52 minutes on average. The discussions were audio-taped. Two observers took additional discussion notes, as well as non-verbal information considered of importance. Furthermore, in order to document the impact of living conditions within the farm, the study collected 64 dietary interviews (24 hour recall) of adult men and women and nine menu registries from two of the farms’ dining halls. Based on the Dietary Guidelines for Americans (15) we categorized food consumption by type and nutritional content, in order to have a qualitative examination of diet adequacy.Descriptive statistics were used to analyze data from individual interviews. Qualitative analysis of group interviews was performed with N Vivo qualitative software 9.0 [15,16]. A dietary database including data from the USDA [16,17] and CIAD [17,18] on regional foods was used to analyze the nutrient supply of jornaleros diets [17].

### 3.2. Results

*Demographics*: Table 3 shows the main characteristics of *jornaleros* in Sonora. The majority of the farmworkers (71.6%) were under the age of 35. Most participants were male (60.5%) and of low income. Fifty three percent (53.2%) percent had a sixth grade education or less, twenty seven percent (27.5%) completed secondary school (middle school) and almost five percent (4.7%) reported some high school or college level education. 

Farmworkers in Sonora reported their birth of origin from several states within Mexico. Most were from the poorest states such as Chiapas, Veracruz, Puebla and Guerrero, according to the human development index (HDI) [18] in these communities, being 0.7336, 0.7754, 0.7929, 0.7513 respectively, compared to a national HDI mean of 0.8031. A lower percentage of farmworkers were born in the northern states of Sonora (HDI, 0.8486) and Sinaloa (HDI 0.811). HDI is calculated using the data on life expectancy, years of formal education and socioeconomic level of families in a community, and as close to 1, these conditions improve.

**Table 3:** Sonora demographics N = 233.

Gender	Male	Female	Total
	n % 141 (60.5)	n % 92 (39.5)	N % 233 (100)
Age			
18–20	29 (20.6)	27 (29.3)	56 (24.0)
21–34	72 (51.1)	39 (42.4)	111 (47.6)
35–44	16 (11.3)	15 (16.3)	31 (13.3)
45–54	12 (8.5)	8 (8.7)	20 (8.6)
55–64	11 (7.8)	2 (2.2)	13 (5.6)
>65	1 (0.7)	1 (1.1)	2 (0.9)
Place of origin			
Chiapas	67 (47.5)	21 (22.8)	88 (37.8)
Veracruz	24 (17.0)	23 (25.0)	43 (18.5)
Puebla	20 (14.2)	10 (10.9)	34 (14.6)
Others	30 (21.3)	38 (41.3)	68 (29.2)
Education			
<6th grade	70 (47.0)	54 (64.3)	124 (53.2)
Secondary 1–3	46 (30.9)	18 (21.4)	64 (27.5)
Preparatory 1–3	10 (6.7)	1 (1.2)	11 (4.7)
Missing	23 (15.4)	11 (13.1)	34 (14.6)
Type of migration			
Pendular with one crop	80 (56.7)	41 (44.6)	121 (51.9)
Golondrino-moving to a variety of crops	39 (27.7)	18 (19.6)	57 (24.5)
Settled (migrants living in Sonora for 4 years or more)	19 (13.5)	28 (30.4)	47 (20.2)
Other	3 (2.1)	5 (5.4)	8 (3.4)
Seguro Social-health insurance			
Yes	35 (27.8)	17 (21.0)	52 (22.3)
No	91 (72.2)	64 (79.0)	155 (66.5)
Missing	16 (12.7)	10 (12.3)	26 (11.2)

In Mexico, migrant farmworkers are hired in their communities (in southern Mexico) by an intermediary (*enganchador*, *intermediario*) and transported to farms in Sonora (northern Mexico) by buses paid in several ways: by intermediary, by the farm owner or in some cases by the Secretaría del Trabajo (Department of Labor). The majority of the samples (51.9%) were pendular stream migrants who go back and forth between their state of origin and Sonora with 25% of the sample moving from crop to crop in various states beyond Sonora and the state of origin. Twenty percent (20%) of the sample was settled out migrants who now live year round in Sonora.

The study focused on migrant farmworkers who were working in grape (69.1%) and vegetable (30.1%) farms in Sonora between April and November of 2007. 

*Health*: As seen in Table 3, three quarters of the participants in the survey did not report any kind of health care insurance. Moreover, for those who had health care coverage it was difficult to access health services. The most common reasons were distance between farms and clinics, lack or cost of adequate transportation and waiting at the clinic. As shown in Table 4, fifty seven percent (133) of those surveyed reported some kind of illness while working in the farm. The most frequently reported diseases included respiratory and intestinal infections as well as injuries caused by work accidents. Infectious diseases continue among the main causes of mortality among indigenous people in Mexico, as this population has the highest levels of poverty within the country [18]. Diabetes, cancer and hypertension were reported by only 3% of the sample among farmworkers in Sonora; however, prevalence may be underreported due to lack of periodic screenings among this population. 

**Table 4:** Sonora farmworker health.

Gender	Male n % 140 (60)	Female n % 93 (40)	Total N % 233 (100)
Reported any disease	80 (51.6)	53 (67.9)	133 (100)
Infectious ^1^			
Gastrointestinal	22 (14.2)	20 (25.6)	42 (18)
Respiratory	44 (28.4)	19 (24.4)	63 (27)
Others (dehydration, headache)	1 (0.6)	4 (5.1)	5 (2.1)
Chronic illness	2 (1.3)	2 (2.6)	4 (1.7)
(Diabetes, cancer and hypertension)			
Anemia	0 (0)	2 (2.6)	2 (0.9)
Work Injuries	6 (3.9)	2 (2.6)	8 (3.4)
Musculoskeletal injuries	5 (3.2)	4 (5.1)	9 (3.9)
Other related to pregnancy	0 (0)	1 (1.3)	1 (0.4)
Anemia	0 (0)	2 (2.6)	2 (0.9)
Nutrition (servings per day)			
Vegetables	1.3	0.5	1.8
Fruits	2.3	1.4	3.7
Wheat tortilla	10.5	5.4	15.9
Corn tortilla	9.5	4.7	14.2
High energy beverages	5.2	3.4	8.6

^1^ Total percentage is higher than 100, since a woman reported both, gastrointestinal and respiratory infections.

The Sonora study also examined a number of environmental determinants that impact health in this population of farmworkers and their families. Our data revealed that overcrowded living conditions were a serious health-related issues, which can be related to infectious diseases within this population. For those living in family housing (usually one to two rooms), roughly 5 to 11 individuals were found sharing sleeping quarters in one room that measured 9 square feet. In addition, individual farmworkers sleeping in large overcrowded barrack-style structures increased their likelihood of transmissible diseases. During the study period, we found between 30 to 60 individuals per living quarter. Each room was fitted with three tiers of small cement beds stacked one above the other.

While the environmental conditions proved inadequate, the nutritional status of farmworkers is just as inauspicious [19]. Mostly high calorie and non-nutritious diets are common, which predisposes farmworkers and their families to obesity and chronic diseases, especially for those who become permanent residents of northern agricultural regions. Data from this study uncovered similar results.

In our study we learned that growers managed dining halls (*comedores*) where meals were prepared on site for farmworkers. Thirty percent (30%) of farmworkers (N = 153) reported that these meals were insufficient in satisfying their hunger or caused illness due to food intolerance or spoiled food. Diets consisted mainly of a combination of wheat (20%) and corn tortillas (80%), fried eggs, rice, chicken soup, tomatoes, cut meats, carbonated and sugary beverages, and coffee. In some cases, when the work is intense (as during the harvest seasons) energy intake is inadequate considering the high caloric expenditure characteristic of agricultural labor. This may be a risk to health among workers performing intensive activities such as the picking grapes. The quality of their diets is deficient as well. Sixty-four percent of total energy comes from simple (mainly sugared beverages and chips) and complex (beans, corn tortilla) carbohydrates, 13% from protein and 22% from fat. Although this appears to abide by internationally recommended standards for carbohydrates and fat, protein intake is low. On the other hand, diets are also low in vitamins A, C, and E and calcium. Tortillas made of corn meal are enriched with iron, zinc and folic acid, which allows for adequate intake of these micronutrients. It is important to note, however, the majority of protein and iron these families consume comes from vegetable sources, which compromises bioavailability and quality. 

In group interviews the workers reported very deficient working conditions and on repeated occasions utilized the expression “we have come to suffer” (*venimos a sufrir*) to describe their daily lives. At the same time they spoke of the necessity to seek out this work as in their home regions there were no jobs or forms of employment. In terms of their living conditions they spoke of overcrowding, high costs of food and drinks, monotonous diets and difficulty adapting to menus from a different region of the country. 

In relation to access to health care in the camps where doctors are on site, the workers felt that the medical staff were poorly trained but were helpful when dealing with usual, routine situations such as diarrhea and respiratory infections. When a doctor was not available transportation difficulties resulted in slower access to care and in emergencies added complexity to an already difficult situation. In general the workers reported self medicating and delaying consulting with health providers considering that the time involved meant a day of lost wages as well as additional cost of transportation. Workers associated diarrhea with changes in the diet provided in the dining halls and in some cases attributed the diarrhea during the grape harvest to the fact that some camps required that the worker constantly taste the product to see if it was ready for harvest. They also reported inadequate housing in terms of being too cold during the winter season resulting in respiratory illnesses.

## 4. Discussion

The results from both studies illustrate the multiple health problems and risk factors experienced by *jornaleros agrícolas* in Sonora mirroring those of migrant farmworkers in Arizona. In terms of demographics there are some important contrasts between the two populations. The Arizona workers are older with less than 18% of the workers between the ages of 21 and 34 compared to 60% of the workers in Sonora between the ages of 21 and 34. While both groups had high percentages of workers with less than a sixth grade education, in Arizona the percentage was 71.9% compared to 62.3% in Sonora. The Sonora sample had a slightly higher percentage of workers with secondary or high school education. Although there is no direct comparison in terms of poverty levels, the Arizona sample shows 76% of the sample with an annual family income of under $25,000 and the majority of the Sonora population coming from the poorer states in Mexico including Chiapas, Veracruz and Puebla. 

Access to health care is an important issue in both populations with less than 52% covered under the health care system of the “Seguro Social” and less than 54% of the Arizona sample having health care insurance in the United States. The Arizona sample had higher percentages of chronic diseases and musculoskeletal injury, which is most likely reflective of the age difference. In contrast, Sonoran workers more often reported respiratory infections and diarrhea with very little chronic disease. The low levels of chronic disease may be due in part to a lack of screening for chronic disease in this population. Sonoran farmworkers perceive the acute infectious illnesses as directly related to working and living conditions. Both populations report low rates of consumption of fruits and vegetables. 

Stress and depression as documented in surveys, the focus groups and group interviews in both populations are also likely contributors to poor health status of the farmworker population. As such stress should be considered within the milieu of challenges to farmworker health. According to the National Institute for Occupational Safety and Health (NIOSH), work-related stress can be caused or exacerbated by long hours and shift work, a poor social environment, lack of opportunity for growth, and unpleasant or dangerous physical conditions [20]. All of these factors apply to agricultural labor and relate to reports documented in our study. NIOSH also reports, stress on the job can increase the likelihood of cardiovascular disease, musculoskeletal disorders, psychological disorders, and workplace injury, as well as suicide, cancer, ulcers, and impaired immune function. 

Racial and ethnic discrimination, working and living conditions in Arizona, working, living conditions and perceptions of abusive treatment by employers in Sonora were documented in the focus group findings as a major sources of stress. This has larger repercussions for their overall health status. Studies have found that racial/ethnic discrimination is commonly linked to poorer mental health status, and can be associated with deteriorated physical health as well [21]. In some cases, there appears to be a direct relationship between racial discrimination and morbidities, though researchers also recognize that health behaviors can be the pathway through which racism negatively impacts health. The majority of studies in this area have focused on Black-White health disparities in the US and immigrant groups in other countries; there is a need to take a closer look at the issue with Mexican migrant and seasonal farmworkers. 

It is clear that the combination of the stress in the daily working and living environment, low educational levels, high poverty levels, and the lack of access to preventive and routine health care are issues that are faced by both populations. Poor diet is also an issue facing this population. At the social and political levels, a combination of supportive policies and social change need to take place in order to combat all of these factors and their ill effects on the population. The economic hardship driving the poor in Mexico to migrate north for work is the same that motivates them to cross the border. Extremely low wages and poor working conditions in Mexico increase farmworkers’ tolerance of conditions in the US, which are poor by national standards but considerably better than those in Mexico. The similarities of *jornaleros agrícolas* and migrant farmworker populations, and the parallel issues they face, speak to a need for a binational response that includes increased social responsibility and attention to social factors and labor conditions as the following proposes:

**(1) Binational Agricultural Labor Standards: **The institutionalization of agricultural labor standards on both sides of the border is a first step toward improving farmworkers’ lives. Like industrial laborers, farmworkers should be able to benefit from standardized, livable wages, safe working conditions, health care, and other benefits. Accessible health care alone would serve to alleviate many other life challenges faced by the population, including stress, poverty, and unemployment. A model of social responsibility in agribusiness can facilitate these changes, but it must be embraced by business leaders. Those who have embraced the process in Mexico or the US can serve as model for others, ultimately increasing productivity thereby demonstrating the value of the approach. A binational agreement or an accord between border states would be a key element to reform working conditions.
**(2) Moving toward a model of social responsibility: **Recommendations emerging from the Sonora project include implementation of a model of corporate social responsibility with agricultural companies, in order to institutionalize better working conditions and benefits for farmworkers. New organizational efforts in the Yuma area are also looking to build partnerships with agribusiness and develop “codes of conduct” to be mutually agreed upon by workers and employers. Working conditions and health issues are only a few of the environmental stressors for farmworkers in the US and Mexico, but the potential for implementing positive change within agribusiness holds promise for improving farmworkers’ health and development.
**(3) Creating Binational Networks: **Educational, health and social service agencies are working to assist migrant farmworkers on both sides of the border, but there is little communication or collaboration between them. Since migrant farmworker populations in the US and Mexico have similar demographic profiles and face parallel challenges, there is a benefit to be had in cross-border partnerships. The establishment of a binational network for farmworker health and development would allow agencies to share information, strategies, materials, and resources to enrich their services and advocate more effectively for their clients.

## 5. Study Limitations

Although the two studies are mainly descriptive, the mixed methods approach strengthened our discussion. Enrollment of workers in study 2 was voluntary and in accordance with the international Review Board policies for research with human subjects, the research team observed the non-discriminatory and voluntary participation policies. Sampling these migrant populations is challenging given they are constantly migrating between regions and farms. Moreover, they live and work in private enterprises whose owners are often reluctant to participate in studies investigating the living conditions of their workers. Accounting for these sampling conditions we can assume that there must be a sampling bias that is inherent to a very mobile population.

## 6. Conclusions

Migrant farmworkers in the US and Mexico live outside of mainstream society. In the US, the legal status of the population tends to be a mix of visa holders, permanent legal residents, the undocumented, and some US citizens. In northern Mexico they are increasingly an indigenous population coming from southern and central Mexico. In both cases they are marginalized from benefits and services available to most people in both countries. The health issues faced by farmworkers, such as diabetes, mirror those of the poor nationally, but farmworkers are unable to access health services they may qualify for due to cultural and language barriers, rural status, and transitory lifestyles. In the US there is the added element of anti-immigrant sentiment and hostilities along the border affecting the mental and physical health of the population, a phenomenon that is only beginning to be understood. The latest legislation involves the state of Arizona. The Arizona legislature recently passed SB 1070, which gained national attention. This legislation requires law enforcement agencies and officials of the state of Arizona to assist in enforcing federal immigration laws. It also grants county attorneys the power to subpoena when investigating employers that hire undocumented workers among other requirements [22]. In Sonora, discrimination due to ethnicity and physical appearance is frequent to people coming from southern Mexico, and *jornaleros* are aware of being discriminated during their visits to health care facilities. It is essential to recognize and analyze the complex needs and special conditions of farmworkers in order to develop programs and policies that afford them dignity and policies that raise their standard of living, which comes with earning a livable wage.
